# Carotid artery assessment in dual-source photon-counting CT: impact of low-energy virtual monoenergetic imaging on image quality, vascular contrast and diagnostic assessability

**DOI:** 10.1007/s11547-024-01889-6

**Published:** 2024-09-17

**Authors:** Christian Booz, Giuseppe M. Bucolo, Tommaso D’Angelo, Silvio Mazziotti, Ludovica R. M. Lanzafame, Ibrahim Yel, Leona S. Alizadeh, Leon D. Gruenewald, Vitali Koch, Simon S. Martin, Mirela Dimitrova, Aynur Goekduman, Thomas J. Vogl, Hanns L. Kaatsch, Daniel Overhoff, Stephan Waldeck

**Affiliations:** 1 Division of Experimental Imaging, Department of Diagnostic and Interventional Radiology, University Hospital Frankfurt, Theodor-Stern-Kai 7, 60590 Frankfurt Am Main, Germany; 2 Department of Diagnostic and Interventional Radiology, University Hospital Frankfurt, Theodor-Stern-Kai 7, 60590 Frankfurt Am Main, Germany; 3 Diagnostic and Interventional Radiology Unit, BIOMORF Department, University Hospital “Policlinico G. Martino”, Via Consolare Valeria 1, 98100 Messina, Italy; 4 Department of Radiology and Nuclear Medicine, Erasmus MC, Doctor Molewaterplein 40, 3015 GD Rotterdam, The Netherlands; 5 Department of Radiology and Neuroradiology, Bundeswehr Central Hospital, Rübenacher Straße 170, 56072 Koblenz, Germany; 6 Department of Radiology and Nuclear Medicine, Medical Faculty Mannheim, University Medical Centre Mannheim, Heidelberg University, Theodor-Kutzer-Ufer 1-3, 68167 Mannheim, Germany; 7 Department of Neuroradiology, University Medical Center Mainz, Langenbeckstraße 1, 55131 Mainz, Germany

**Keywords:** Carotid arteries, Virtual monoenergetic imaging, Photon-counting CT, Dual-energy CT

## Abstract

**Purpose:**

Preliminary dual-energy CT studies have shown that low-energy virtual monoenergetic (VMI) + reconstructions can provide superior image quality compared to standard 120 kV CTA series. The purpose of this study is to evaluate the impact of low-energy VMI reconstructions on quantitative and qualitative image quality, vascular contrast, and diagnostic assessability of the carotid artery in patients undergoing photon-counting CTA examinations.

**Materials and methods:**

A total of 122 patients (67 male) who had undergone dual-source photon-counting CTA scans of the carotid artery were retrospectively analyzed in this study. Standard 120 kV CT images and low-keV VMI series from 40 to 100 keV with an interval of 15 keV were reconstructed. Quantitative analyses included the evaluation of vascular CT numbers, signal-to-noise ratio (SNR), and contrast-to-noise ratio (CNR). CT number measurements were performed in the common, external, and internal carotid arteries. Qualitative analyses were performed by three board-certified radiologists independently using five-point scales to evaluate image quality, vascular contrast, and diagnostic assessability of the carotid artery.

**Results:**

Mean attenuation, CNR and SNR values were highest in 40 keV VMI reconstructions (HU, 1362.32 ± 457.81; CNR, 33.19 ± 12.86; SNR, 34.37 ± 12.89) followed by 55-keV VMI reconstructions (HU, 736.94 ± 150.09; CNR, 24.49 ± 7.11; SNR, 26.25 ± 7.34); all three mean values at these keV levels were significantly higher compared with the remaining VMI series and standard 120 kV CT series (HU, 154.43 ± 23.69; CNR, 16.34 ± 5.47; SNR, 24.44 ± 7.14) (*p* < 0.0001).

The qualitative analysis showed the highest rating scores for 55 keV VMI reconstructions followed by 40 keV and 70 keV VMI series with a significant difference compared to standard 120 kV CT images series regarding image quality, vascular contrast, and diagnostic assessability of the carotid artery (all comparisons, *p* < 0.01).

**Conclusions:**

Low-keV VMI reconstructions at a level of 40–55 keV significantly improve image quality, vascular contrast, and the diagnostic assessability of the carotid artery compared with standard CT series in photon-counting CTA.

## Introduction

The carotid arteries, playing a crucial role in supplying blood to the head, face, and neck, are often susceptible to a diverse spectrum of pathological conditions, including stenosis, dissection, and pseudoaneurysm. Atherosclerotic disease is the main cause of carotid artery stenosis and the leading contributor of stroke and transient ischemic attack (TIA), accounting for approximately 15% of all ischemic strokes and TIAs [1, 2]. Notably, also asymptomatic patients can be affected by carotid artery atherosclerosis: In fact, approximately 3% of men and 1% of women over 80 years of age show severe stenosis [2]. On the contrary, in younger patients, carotid artery dissection is the most frequent pathology, which can result in an early stroke, with a prevalence of 20–25% in patients less than 45 years of age [3, 4].

CT angiography (CTA) represents the non-invasive method of choice in acute cases and for accurate carotid and intracranial cerebral vessel assessment [5, 6]. To achieve optimal arterial opacification with minimal venous contamination, the contrast media protocol and the scanning time have to be carefully optimized. Similar to CTA performed on other body regions, achieving optimal visualization of supra-aortic arteries, it requires accurate and precise timing to maximize the best contrast signal, which is dependent on contrast medium, CT scanner, and certain patient-related factors.

In recent years, dual-energy CT (DECT) has provided numerous benefits in vascular imaging due to technical developments such as virtual monoenergetic imaging (VMI) [7, 8], virtual non-contrast (VNC) series and iodine mapping [9, 10], improving image quality and the diagnostic accuracy for various pathologies in clinical routine [11].

Particularly 40–55 keV VMI reconstructions demonstrated improving image contrast in vascular assessment, which could lead to a reduction of contrast media dose [12].

The novel photon-counting CT (PCCT) technique represents a cutting-edge advancement in imaging, building upon the evolution of DECT [13]. This technique provides numerous advantages compared with both conventional CT and DECT. It improves spectral resolution and material decomposition, enables faster scanning times, and reduces radiation dose and image noise through the application of new specialized detectors, made of cadmium telluride, able to directly measure the energy of individual X-ray photons [14]. Up to date, PCCT has been employed in measuring components in excised atherosclerotic plaques, obtained from carotid endarterectomy compared to histopathology. Dahal et al. [15] demonstrate the potential of PCCT for identifying high-risk carotid plaque features accurately, suggesting promising implications for improving cardiovascular risk stratification. However, limited studies are available evaluating the use of PCCT low-keV VMI specifically for carotid artery assessment to the best of our knowledge. Thus, our aim was to assess the impact of low-keV VMI PCCT reconstructions on quantitative and qualitative image quality, vascular contrast, and diagnostic assessability of the carotid artery in patients undergoing photon-counting CTA examinations.

## Material and methods

This retrospective study was approved by our institutional review board, and informed consent to radiological examination was obtained.

### Study population

**Fig. 1:**
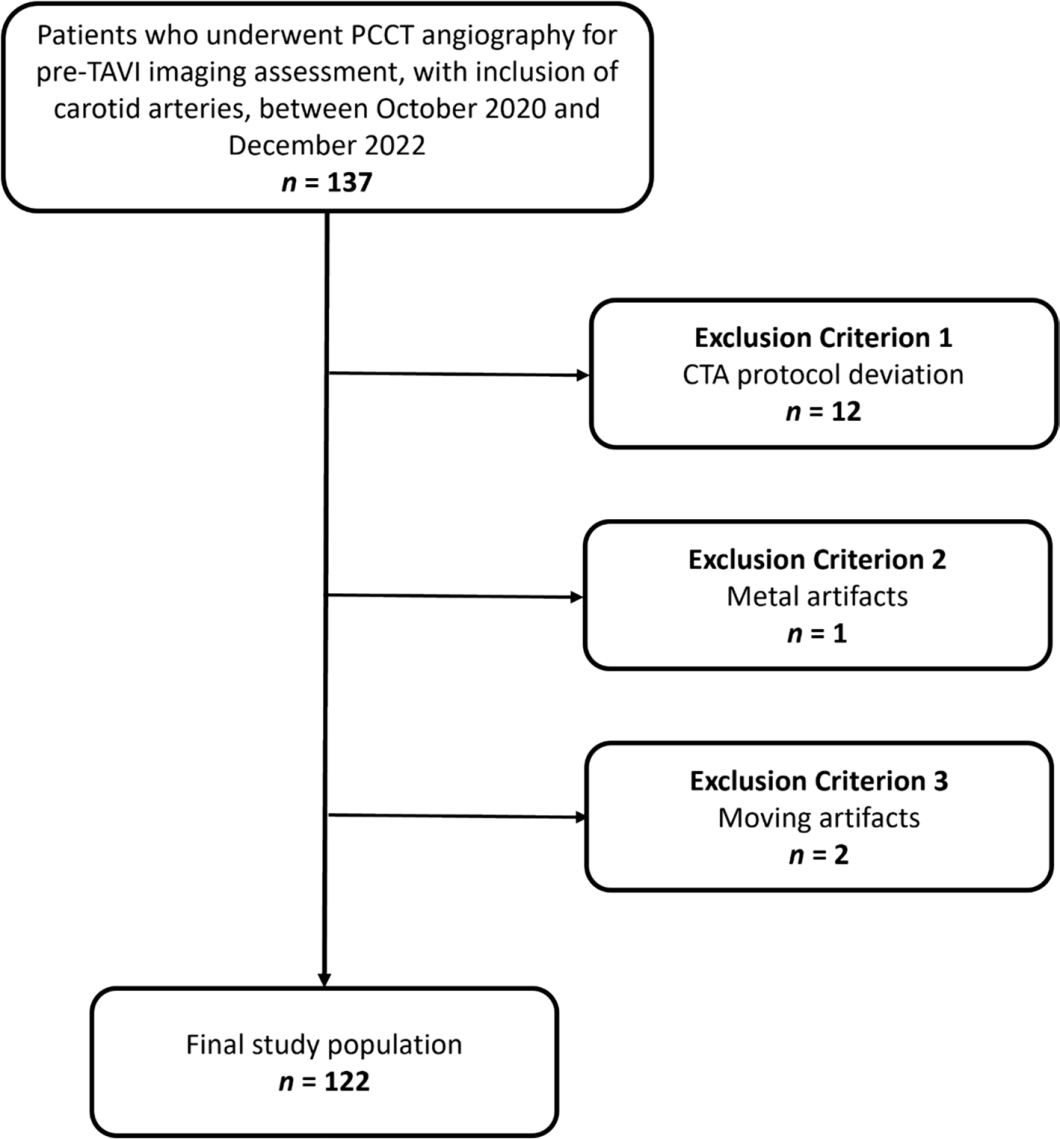
Flowchart showing the selection process in this study of patient inclusion and exclusion criteria. *PCCT* Photon-counting CT; *CTA* CT angiography

We reviewed our institutional databases to identify patients who underwent contrast-enhancement PCCT angiography including carotid vessels between October 2020 and December 2022 prior to transcatheter aortic valve implantation (TAVI). We excluded any patients deviating from the standard CTA protocol (n = 12). Further, we excluded three patients due to metal artifacts (n = 1) and moving artifacts (n = 2) affecting carotid vessels. The study inclusion process is displayed in the flowchart in Fig. 1.

### CT Scan Protocol

All scans were performed on a dual-source PCCT scanner (NAEOTOM Alpha, Siemens Healthineers, Forchheim, Germany). Patients were examined in supine position, with their arms by their side. Preliminary, scout scans in anteroposterior and lateral directions were acquired, ranging from the neck to the iliac vessels in context of TAVI planning.

The study protocol included an unenhanced ECG-triggered scan of the heart, followed by intravenous administration of contrast medium (Xenetix 350 mgI/ml, Guerbet, Villepinte, France) for the angiographic phase. 80 mL contrast agent was injected though a superficial vein of the forearm with a flow rate of 5 mL/s, followed by a bolus of 50 ml of saline at mL/s, using CT Motion (Ulrich Medical, Ulm, Germany) power injector. The angiographic phase was started bolus triggered (region of interest in the descending aorta) with an ECG-synchronized CTA of the neck, thorax, abdomen, and pelvis (from carotid vessels to femoral arteries) and a pitch of 3.2. The start of the acquisition was chosen automatically to ensure a data acquisition of the aortic valve at 40% RR interval.

### Image reconstruction

All data were sent to a dedicated postprocessing workstation (syngo.via V70, Siemens Healthineers, Forchheim, Germany). The spectral postprocessing datasets were analyzed with a slice thickness of 1.0 mm, and an increment of 1.0 mm was reconstructed using a soft tissue convolution kernel (Qr40; Siemens).

Using a dedicated postprocessing workstation (syngo.via version VB60A, Siemens Healthineers, Forchheim, Germany), VMI series were reconstructed at different virtual energy levels from 40 to 100 keV in 15 keV increments using the dedicated postprocessing software. Moreover, standard images equivalent to single-energy polychromatic 120 kV acquisition were reconstructed.

### Quantitative image analysis

One radiologist with 5 years of experience in CTA drew a total of 10 ROIs with a range size of 3 mm^2^ to 20 mm^2^ within the lumen of the common carotid artery (CCA), external carotid artery (ECA), internal carotid artery (ICA), in both sides (5 per side). All ROIs were positioned covering the largest possible lumen and avoiding vessel borders, calcified and non-calcified plaque, equal for each reconstruction.

Attenuation values (HU) and standard deviation (SD) were extracted for each reconstructed series. The average values of the measurements were used for statistical analyses.

Attenuation values and SD measurements of subcutaneous fat and psoas muscle were recorded for the evaluation of signal-to-noise ratio (SNR) and contrast-to-noise ratio (CNR), calculated using the following formulas:

$$ SNR = \frac{{{\text{HU}}_{{{\text{artery}}}} }}{{{\text{SD}}_{{{\text{fat}}}} }}; \;{\text{CNR}} = \frac{{\left( {{\text{HU}}_{{{\text{artery}}}} {-} {\text{HU}}_{{{\text{muscle}}}} } \right)}}{{{\text{SD}}_{{{\text{fat}}}} }} $$

The SD of subcutaneous fat was used as the reference of image noise.

### Qualitative image analysis

Three board-certified radiologists (*BLINDED* with 6–10 years of experience in vascular imaging) evaluated VMI reconstructions from 40 to 100 keV and the standard series. Image evaluation was performed with a conventional picture archiving and communication system workstation (Centricity, version 4.2; GE Healthcare, Solingen, Germany). The used criteria were the overall image quality using a five-point scale from 1 to 5 (1 = very poor, 5 = optimal), vascular contrast (1 = very poor, 5 = optimal), and diagnostic assessability of the carotid artery (1 = non diagnostic, 5 = optimal) (Table 1). Each reader was free to adjust window settings and scroll through the whole stack of each CT series.

**Table 1:** spsp

	Overall Image Quality	Vascular contrast	Diagnostic assessability
1	Very poor	Very poor	Not diagnostic
2	Poor	Poor	Poor
3	Acceptable	Acceptable	Acceptable
4	Good	Good	Good
5	Optimal	Optimal	Optimal

Five-point Likert Scale for subjective analysis of image quality, vascular contrast, and diagnostic assessability

### Statistical analysis

Statistical analysis was performed using statistical software (MedCalc; MedCalc Software, Ostend, Belgium).

Data distribution was evaluated using the Kolmogorov–Smirnov test. The comparison of values between each VMI reconstruction was assessed using repeated measures analysis of variance test for normal distribution data, and with Friedman test for non-normal data.

Fleiss’ kappa was used to assess the reliability of agreement between readers. A kappa statistic between 0.61 and 0.8 was rated as substantial agreement, and above 0.81 as almost perfect agreement. A Kappa of 0 meant poor, between 0 and 0.2 slight, 0.21–0.4 fair, and 0.41–0.6 moderate agreement [16].

The statistically significant difference was indicated by a *p-* value less than 0.05.

All continuous variables are shown as mean and standard deviation (SD).

## Results

### Population characteristics

The final cohort of our study included 122 patients, aged 78.10 ± 10.0 years (range 59–92 years). The cohort consisted of 62 males, aged 76.9 ± 11.8 years (range 59–90 years), and 60 females, aged 79.6 ± 8.1 years (range 61–92 years), all of whom underwent PCCT prior to TAVI. The mean body mass index (BMI) of the patients was 29.4 ± 4.3 kg/m^2^, with a range of 28.5 to 32.8 kg/m^2^. Patients also present the following diagnosed comorbidities: hypertension (96; 79%), mellitus diabetes (65; 53%), peripheral arterial disease (46; 38%), coronary artery disease (81; 66%), and severe kidney insufficiency (15; 12%). Table 2 shows all patient characteristics.

**Table 2:** bbbb

Characteristic	All Patients (n = 122)
Sex, no. (%)	
*Male*	62 (51%)
*Female*	60 (49%)
Age, mean ± SD (range)	
*Male*	63.9 ± 11.8 (42–84)
*Female*	62.7 ± 8.1 (39–73)
BMI, mean ± SD (range)	29.4 ± 4.3 kg/m^2^ (28.5–32.8 kg/m^2^)
Diagnosed Comorbidities	
*Hypertension*	96 (79%)
*Mellitus Diabetes*	65 (53%)
*Peripheral arterial disease*	46 (38%)
*Coronary artery disease*	81 (67%)
*Severe Kidney Insufficiency*	15 (12%)

Patient characteristics

*BMI* Body mass index

All included examinations adhered to the examination protocols without any complications, and no examination in this cohort had to be repeated. The mean cumulative CT dose index (CTDIvol) across all examinations was 5.96 ± 2.23 mGy. The mean cumulative dose length product (DLP) was recorded as 398.3 ± 189.2 mGy cm.

### Quantitative image analysis

Attenuation values were highest in the 40 keV VMI series compared to the other VMI and standard 120 kV reconstructions (*p* < 0.0001), with all vessels’ average value of 1362.32 ± 457.81 HU, while standard 120 kV had a value of 154.43 ± 23.69 HU. Subsequently, image noise at 40-keV VMI series was 40.46 ± 5.74, the highest compared to other reconstructions.

**Fig. 2:**
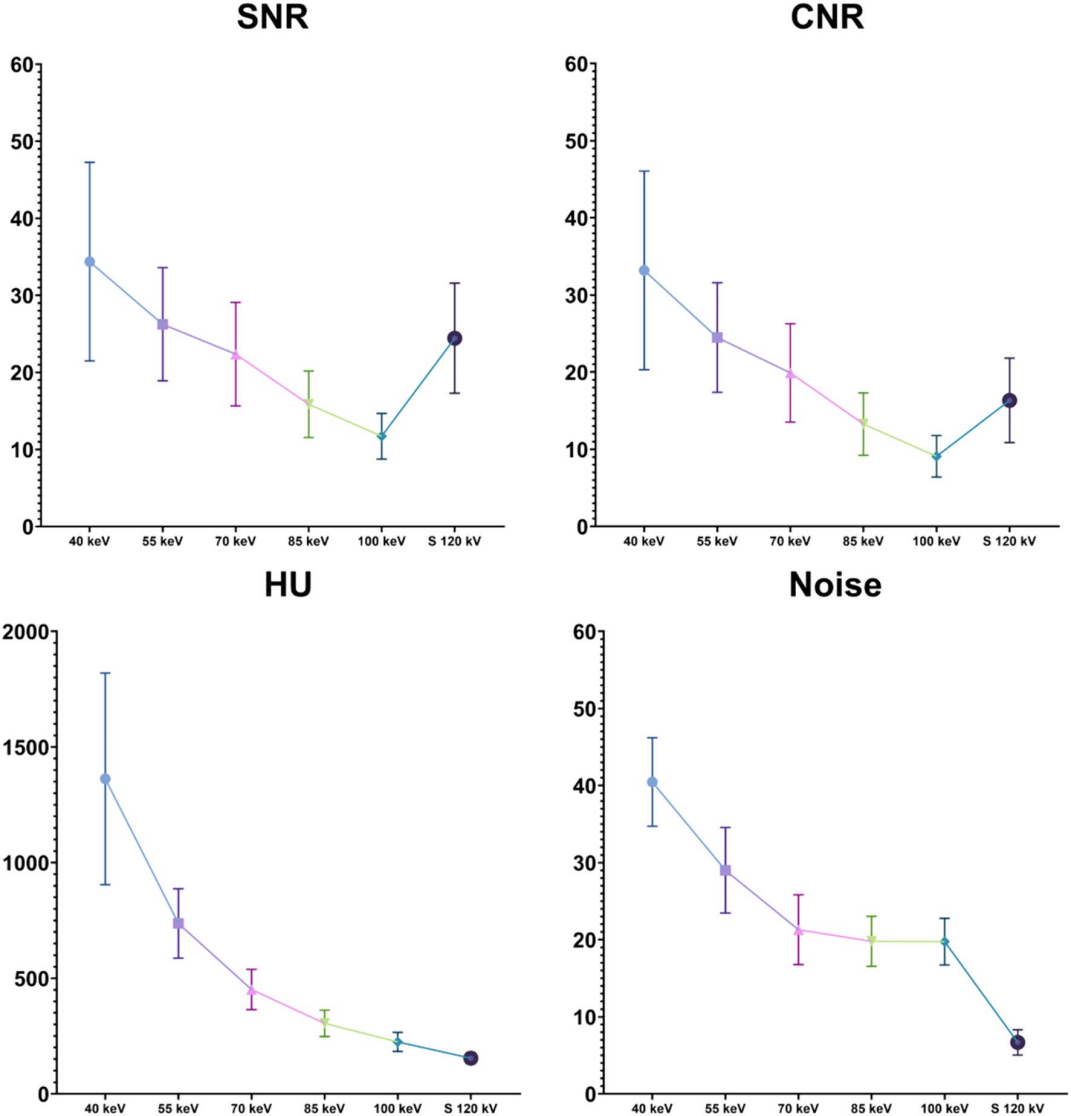
The graphs show distribution of SNR, CNR, attenuation value (HU) and image noise at various keV levels (from 40 to 100 keV) and standard 120 kV series for all the vessels. The highest values for all the parameters are demonstrated in 40-keV VMI, with mean values at 40 keV of SNR (34.37 ± 12.89), CNR (33.19 ± 12.86), attenuation values (1362.32 ± 457.81 HU), and image noise (40.46 ± 5.74). Attenuation values and image noise gradually decrease as the energy level (keV) increases. *SNR* Signal-to-noise ratio; *CNR* Contrast-to-noise ratio; *HU* Hounsfield unit; *S* Standard

Moreover, calculated SNR and CNR were significantly higher in the 40-keV VMI series compared to the other VMI reconstructions (*p* < 0.0001), with all vessels’ average value of 34.37 ± 12.89 and 33.19 ± 12.86, respectively (Fig. 2). All the quantitative measurements are displayed in Table 3.

**Table 3:** yyyy

Parameters	40 keV	55 keV	70 keV	85 keV	100 keV	Standard 120 kV	*p* value
SNR							
CCA	34.29 ± 9.80	26.78 ± 7.58	22.85 ± 7.15	16.27 ± 4.54	11.98 ± 3.04	25.15 ± 6.85	< 0.0001
ECA	32.33 ± 8.60	25.28 ± 7.06	21.67 ± 6.42	15.38 ± 4.03	11.41 ± 2.79	23.25 ± 7.42	< 0.0001
ICA	26.50 ± 17.96	26.69 ± 7.31	22.58 ± 6.58	15.96 ± 4.34	11.77 ± 3.02	24.92 ± 7.74	< 0.0001
AVERAGE	34.37 ± 12.89	26.25 ± 7.34	22.37 ± 6.72	15.87 ± 4.31	11.72 ± 2.95	24.44 ± 7.14	< 0.0001
CNR							
CCA	33.10 ± 9.77	25.02 ± 7.38	20.39 ± 6.80	13.67 ± 4.28	9.35 ± 2.78	17.05 ± 5.02	< 0.0001
ECA	31.15 ± 8.55	23.52 ± 6.82	19.20 ± 6.06	12.78 ± 3.75	8.78 ± 2.53	15.15 ± 5.91	< 0.0001
ICA	35.32 ± 17.94	24.93 ± 7.06	20.12 ± 6.21	13.36 ± 4.06	9.14 ± 7.50	16.82 ± 5.82	< 0.0001
AVERAGE	33.19 ± 12.86	24.49 ± 7.11	19.90 ± 6.37	13.27 ± 4.04	9.09 ± 2.69	16.34 ± 5.47	< 0.0001
HU							
CCA	1358.88 ± 299.82	751.68 ± 160.24	460.91 ± 93.58	312.45 ± 60.79	229.65 ± 43.48	159.19 ± 21.88	< 0.0001
ECA	1282.78 ± 263.18	709.53 ± 138.02	437.53 ± 81.37	295.73 ± 52.06	218.73 ± 39.13	146.99 ± 26.35	< 0.0001
ICA	1445.29 ± 678.06	749.60 ± 148.61	456.38 ± 84.35	306.65 ± 57.44	225.15 ± 40.92	157.12 ± 23.25	< 0.0001
AVERAGE	1362.32 ± 457.82	736.94 ± 150.09	451.61 ± 86.94	340.94 ± 57.14	224.51 ± 41.35	154.43 ± 23.69	< 0.0001
NOISE	40.46 ± 5.74	29.01 ± 5.55	21.30 ± 4.52	19.80 ± 3.24	19.75 ± 3.03	6.68 ± 1.64	< 0.0001

Results of the quantitative measurements in the common carotid artery (CCA), external carotid artery (ECA), and internal carotid artery (ICA). All data are displayed in mean ± SD

*HU* Hounsfield unit; *SNR* Signal-to-noise ratio; *CNR* Contrast-to-noise ratio

**Fig. 3:**
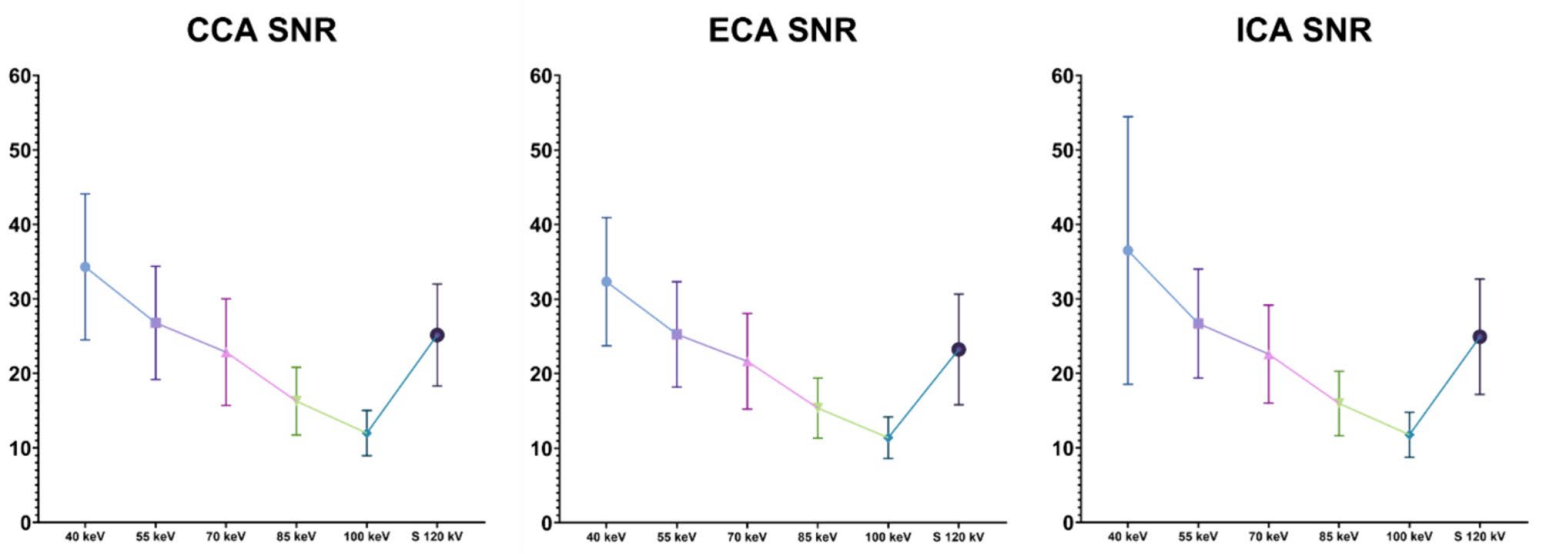
SNR distributions of VMI algorithms at various keV levels (from 40 to 100 keV) and the standard 120 kV series grouped by vessels. The graphs show higher SNR in low-keV reconstruction, especially in 40-keV VMI reconstructions. The mean value of SNR at 40-keV grouped by vessels are CCA 32.29 ± 9.80; ECA 32.33 ± 8.60; ICA 36.50 ± 17.96. *SNR*: Signal-to-noise ratio; *CCA* Common carotid artery; *ECA* External carotid artery; *ICA* Internal carotid artery; *S* Standard

**Fig. 4:**
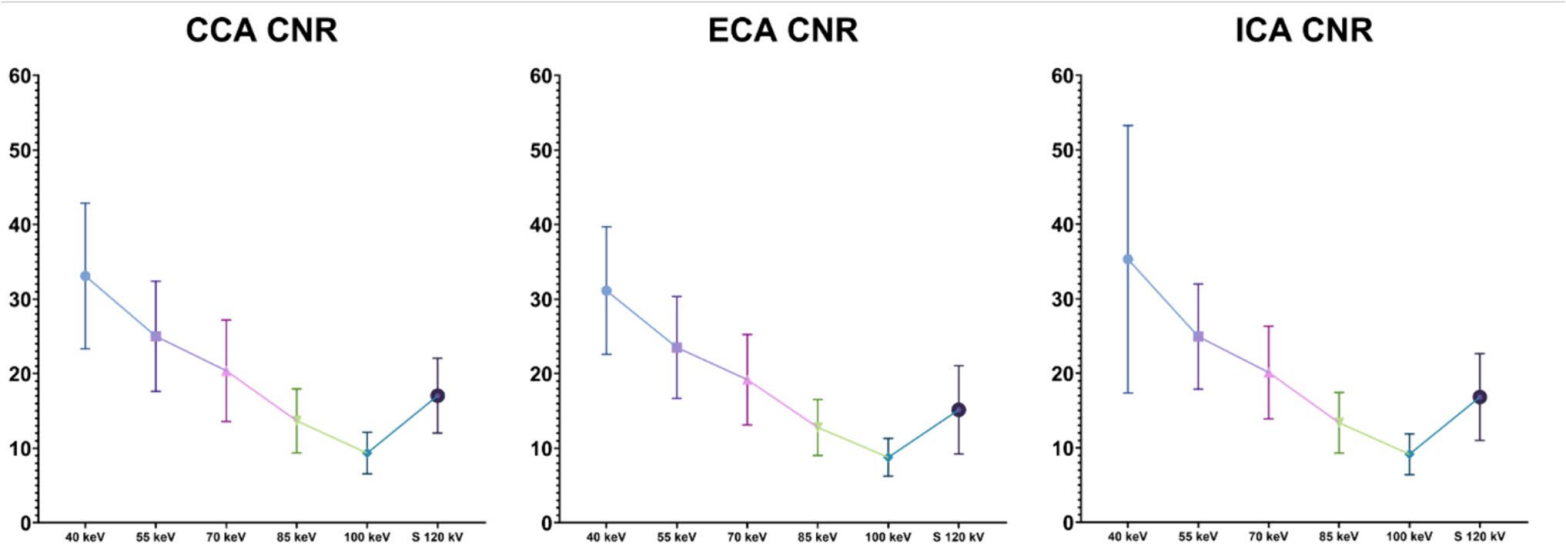
CNR distributions of VMI algorithms at various keV levels (from 40 to 100 keV) and the standard 120 kV series grouped by vessels. The graphs show higher CNR in low-keV reconstruction, especially in 40-keV VMI reconstructions. The mean value of CNR at 40-keV grouped by vessels are CCA 33.10 ± 9.77; ECA 31.15 ± 8.55; ICA 35.32 ± 17.94. *CNR* Contrast-to-noise ratio; *CCA* Common carotid artery; *ECA* External carotid artery; *ICA* Internal carotid artery; *S* Standard

SNR and CNR have shown a progressive reduction with energy levels increasing in VMI reconstructions for each studied vessel (Figs. 3 and 4).

### Qualitative image analysis

Qualitative image quality assessment revealed the highest ratings for the 55 keV VMI reconstructions, with an overall image quality of 4.6 ± 0.3, a vascular contrast rating of 4.4 ± 0.4 and a diagnostic assessability of the carotid artery of 4.2 ± 0.7. There was substantial interobserver agreement for this category, with a median score of 5 and a kappa value of 0.76 (95% CI: 0.58–0.92). This was followed closely by the 70 keV VMI reconstructions with an overall image quality of 4.4 ± 0.4, a vascular contrast rating of 4.2 ± 0.5 and a diagnostic assessability of the carotid artery of 4.0 ± 0.8. The differences between these low-keV VMI series were not statistically significant (*p* > 0.05). In standard polychromatic 120 kV images, the overall image quality was 3.4 ± 0.3, the vascular contrast rating 3.2 ± 0.4 and the diagnostic assessability of the carotid artery was 3.1 ± 0.6.

**Fig. 5:**
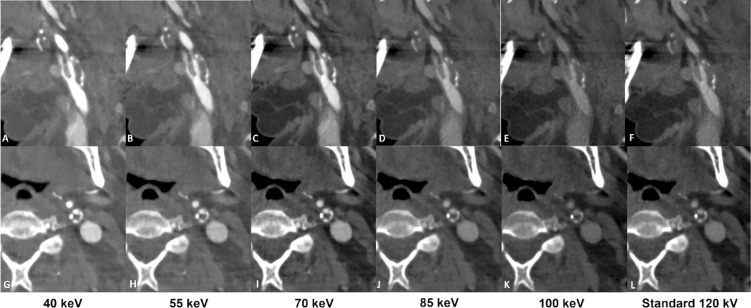
A 76-year-old male suffering from aortic valve stenosis and comorbidities such as diabetes mellitus type 2, and primary arterial hypertension, undergoing PCCT angiography prior to TAVI using 80 ml of 350 mgI/mL Xenetix at an infusion rate of 5.0 ml/second (Xenetix 350 mgI/ml, Guerbet, Villepinte, France). PCCT angiography reconstructions in the parasagittal plane (A-F) and axial plane (G-L) display a subtotal occlusion at the origin of ICA and normal opacification of CCA and ECA. Low-keV VMI + reconstructions ranging from 40 – 70 keV, demonstrate higher iodine attenuation within the perfused lumen and a higher contrast between perfused lumen and soft plaque material compared to the other keV VMI levels and standard CT images. Additionally, beam hardening artifacts are less present in parasagittal reconstructions at low-keV VMI series compared to standard 120 kV images, particularly at 40-55 keV. *CCA* Common Carotid Artery; *ECA* External Carotid Artery; *ICA* Internal Carotid Artery; *PCCT* Photon-counting CT; *TAVI* Transcatheter Aortic Valve Implantation

When comparing across the range of energy levels, VMI images from 40 to 85 keV received superior overall image quality ratings (all *p* < 0.01) compared to those from higher keV images. The results of the subjective image analysis are summarized in Table 4. Figure 5 displays a general impression of the image changes with increasing VMI.

**Table 4:** xxxx

keV	Overall image quality	Vascular contrast	Diagnostic assessability	Kappa value	*P* value
40 keV	4.0 ± 0.4	4.1 ± 0.3	3.6 ± 0.8	0.79 (95% CI 0.62–0.94)	All, *p* < 0.01
55 keV	4.6 ± 0.3	4.4 ± 0.4	4.2 ± 0.7	0.76 (95% CI 0.58–0.92)	55–70 keV, *p* > 0.05 *
					Others, *p* < 0.01
70 keV	4.4 ± 0.4	4.2 ± 0.5	4.0 ± 0.8	0.81 (95% CI 0.69–0.96)	70–55 keV, *p* > 0.05 *
					Others, *p* < 0.01
85 keV	3.2 ± 0.3	3.0 ± 0.5	3.0 ± 0.4	0.79 (95% CI 0.62–0.91)	All, *p* < 0.01
100 keV	2.5 ± 0.6	2.5 ± 0.8	2.0 ± 0.7	0.77 (95% CI 0.64–0.90)	All, *p* < 0.01
Standard 120 kV	3.4 ± 0.3	3.2 ± 0.4	3.1 ± 0.6	0.74 (95% CI 0.51–0.85)	All, *p* < 0.01

Average results of the qualitative assessment of all carotid arteries. All data are displayed in mean ± SD. Bonferroni correction was applied with an alpha level set at 0.05

Asterisk (*) indicates the absence of statistical significance

All *p* values remained unchanged after the correction

## Discussion

Our results show contrast and image quality improvement in low-keV VMI reconstructions in the evaluation of carotid vessels in comparison with other VMI energy levels and standard images in PCCT. In particular, 40 keV VMI showed the highest mean attenuation, CNR and SNR (HU, 1362.32 ± 457.81; CNR, 33.19 ± 12.86; SNR, 34.37 ± 12.89) followed by 55-keV VMI series (HU, 736.94 ± 150.09; CNR, 24.49 ± 7.11; SNR, 26.25 ± 7.34). Furthermore, the low-keV VMI series (40–85 keV) received a superior subjective rating compared to both higher keV images and the standard polychromatic 120 kV. Notably, 55-keV VMI reconstructions exhibited the highest values for subjective parameters, demonstrating an overall image quality of 4.6 ± 0.3. This was closely followed by the 70-keV VMI, with an overall image quality of 4.2 ± 0.7 with not statistically significance (*p* > 0.05). In contrary, subjective image quality assessment yielded superior ratings for 70-keV VMI, possibly due to the visual perception of high noise levels at 40-keV.

As already demonstrated in the literature, DECT-derived VMI + provides numerous advantages in cardiovascular imaging, improving image quality and diagnostic accuracy [17–21]. In particular, low-keV levels (40–75 keV) close to the iodine K-edge (33.17 keV), increase the iodine attenuation, providing superior quantitative image quality parameters, such as SNR and CNR [22]. Previous studies evaluated DECT VMI for vascular assessment. In this context, Leithner et al. [23] demonstrated increased suitability of 40-keV VMI + images for carotid and intracranial artery assessment using a second-generation dual-.source DECT system. Aside from this, low-keV VMI + showed to reduce the contrast medium dose needed to obtain good image quality with high diagnostic value [24, 25].

There are pioneering PCCT studies evaluating VMI for vascular assessment. Dillinger et al. [26] evaluated PCCT VMI on the visualization of abdominal arterial vessels, demonstrating that 60–70 keV VMI provides best results in qualitative and quantitative image quality assessment, which is slightly different compared to our results showing optimal image quality and suitability for carotid artery assessment at lower keV (40–55 keV energy series). Accordingly with our findings, Sartoretti et al. [27] demonstrated the value of PCCT VMI in the evaluation of coronary arteries, with optimal image quality at an energy level of 40 keV. Reason for these results may be the invention of photon-counting detectors, allowing the reduction of electronic noise by counting the number of pulses greater than a preset threshold; secondly, photons of different energy are equally weighted, unlike in DECT technologies with energy-integrating detectors (EID), where high-energy photons contribute to more than low-energy photons, consequently decreasing CNR [28].

**Fig. 6:**
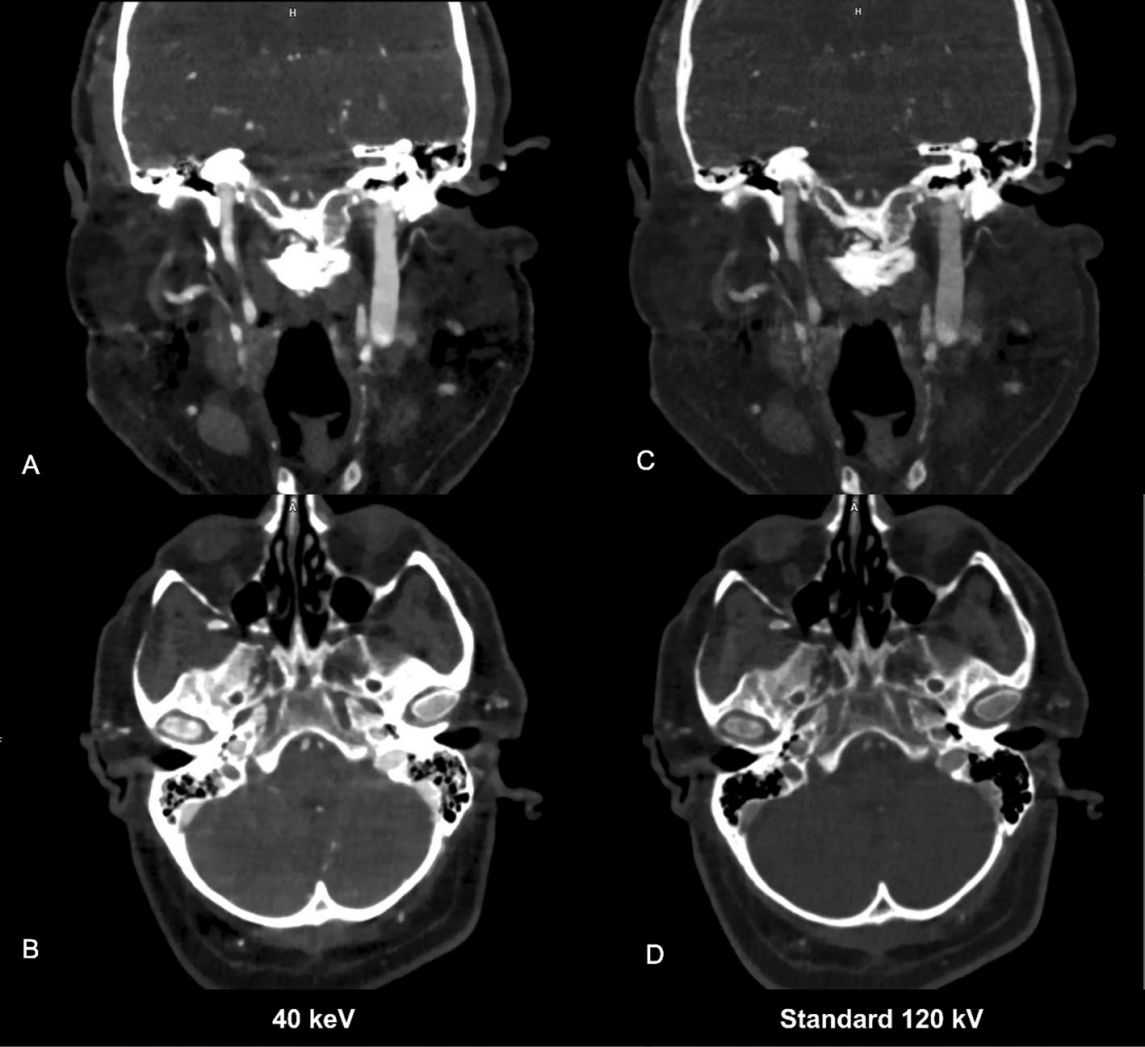
A 78-year-old female with aortic valve stenosis and comorbidities including diabetes mellitus type 2, and chronic kidney disease, undergoing PCCT angiography pre-TAVI, using 80 ml of 350 mgI/mL Xenetix at an infusion rate of 5.0 ml/second (Xenetix 350 mgI/ml, Guerbet, Villepinte, France). PCCT angiography reconstructions in paracoronal and axial plane (**A**–**B**) at low-VMI energy levels 40 keV show less amount of hypoattenuating artifacts and a regular opacification of right ICA, clearly indicating a regular perfusion, compared to paracoronal and axial reconstructions (**C**–**D**) in standard 120 kV. *ICA* Internal Carotid Artery; *PCCT* Photon-counting CT; *TAVI* Transcatheter Aortic Valve Implantation

In addition, the increased iodine attenuation at low keV (40–55 keV) could lead to a reduction of contrast medium amount, still maintaining high image quality for clinical practice. Previous studies conducted with DECT have been presumed to enable a reduction of contrast media amount [22, 29]. This may be very useful in patients with comorbidities, such as mellitus diabetes and renal kidney disease [12, 30]. Although our study did not specifically investigate this aspect, our findings showed a substantially improved quantitative image quality at low keV, far greater than 300 HU average enhancement defined as diagnostic for vascular structures [31, 32]. This improvement suggests the possibility to reduce contrast medium amount, while preserving image quality comparable to that of standard CTA. This significant improvement concerning iodine attenuation could open up possibilities for optimizing acquisition parameters with the aim of reducing radiation dose during CTA scans. Further studies are needed to comprehensively evaluate the feasibility and effectiveness of this future perspective (Fig. 6).

Moreover, PCCT maintains high time resolution with no risk of compromising vascular imaging [33]. Furthermore, PCCT provides spectral imaging in every CT scans. As a result, the integration of PCCT applications into routine clinical practice holds the potential to modify current protocols, allowing reduction of contrast medium dose through VMI, as well as radiation dose, also because of the availability of VNC substituting the native scan in selecting cases. In this context, VNC demonstrated the capability to differentiate between intracranial hemorrhage and extravasation of iodinated contrast media after ischemic stroke therapy with high diagnostic accuracy [34, 35]. This benefit could enable radiologists to use dedicated applications, even for the evaluation of incidental findings, where the lack of a dedicated protocol in conventional CT would otherwise render a definite diagnosis impossible, or in case of insufficient intravascular enhancement (e.g., in case of a missed contrast medium bolus) [36].

This study has limitations that have to be addressed. First, the retrospective study design may have influenced our results. Second, the ROI drawing was difficult in some cases due to vessel disease (calcifications and soft plaques) and may have affected the analyses. Third, we arbitrarily chose energy levels from 40 to 100 keV, with an increment of 15 keV; results may be different at unrated energy levels. Higher energy levels were not included because our study is focused on the benefit of VMI in the improvement of iodine attenuation and image quality in carotid CTA, provided by low-energy level, according to the literature. Nevertheless, it should be noted that in the case of calcified plaques or stenosis, higher VMI energy levels may reduce blooming artifacts. Indeed, we did not evaluate the diagnostic performance in the assessment of carotid pathologies, such as stenosis and atheromatic plaques (both soft and calcified). In this context, standard 120 kV reconstructions play a crucial role in minimizing artifacts arising from extensive calcified components within the plaque. Forth, the relatively small sample size based on available patient data may affect the generalizability of our results; further studies with larger prospective cohorts are needed to provide more robust evidence in this field. Finally, we have not conducted a direct comparison between PCCT technology and older DECT technologies; consequently, the assessment of VMI performance obtained from these two distinct technologies remains unknown.

In conclusion, PCCT 40-keV VMI showed the highest values of SNR and CNR in quantitative evaluation. Conversely, regarding subjective evaluation of vessel assessment, 55-keV VMI received the best scores. Thus, our results suggest that these VMI PCCT reconstructions (40–55 keV) could be feasible to optimize the suitability for carotid arteries assessment, improving image quality, compared to standard 120 kV reconstructions. Moreover, in clinical practice, the ever-available spectral dataset might improve vascular visualization in all studies, even those performed with non-vascular protocols, thereby leading to a reduction in contrast medium amount.

## Data Availability

The full dataset will not be provided for publication at this moment due to further upcoming research projects related to department of University Hospital Frankfurt.

## Abbreviations

CTA: CT angiography
DE: Dual energy
DECT: Dual-energy computed tomography
EID: Energy-integrating detectors
PCCT: Photon-counting CT
ROI: Region of interest
SD: Standard deviation
VMI: Virtual monoenergetic imaging
VNC: Virtual non-contrast
